# EEG-powered cerebral transformer for athletic performance

**DOI:** 10.3389/fnbot.2024.1499734

**Published:** 2024-12-20

**Authors:** Qikai Sun

**Affiliations:** Sports Department of Zhejiang A&F University, Hangzhou, Zhejiang, China

**Keywords:** EEG signals, sports performance analysis, cross-modal fusion, attention mechanism, transformer

## Abstract

**Introduction:**

In recent years, with advancements in wearable devices and biosignal analysis technologies, sports performance analysis has become an increasingly popular research field, particularly due to the growing demand for real-time monitoring of athletes' conditions in sports training and competitive events. Traditional methods of sports performance analysis typically rely on video data or sensor data for motion recognition. However, unimodal data often fails to fully capture the neural state of athletes, leading to limitations in accuracy and real-time performance when dealing with complex movement patterns. Moreover, these methods struggle with multimodal data fusion, making it difficult to fully leverage the deep information from electroencephalogram (EEG) signals.

**Methods:**

To address these challenges, this paper proposes a "Cerebral Transformer" model based on EEG signals and video data. By employing an adaptive attention mechanism and cross-modal fusion, the model effectively combines EEG signals and video streams to achieve precise recognition and analysis of athletes' movements. The model's effectiveness was validated through experiments on four datasets: SEED, DEAP, eSports Sensors, and MODA. The results show that the proposed model outperforms existing mainstream methods in terms of accuracy, recall, and F1 score, while also demonstrating high computational efficiency.

**Results and discussion:**

The significance of this study lies in providing a more comprehensive and efficient solution for sports performance analysis. Through cross-modal data fusion, it not only improves the accuracy of complex movement recognition but also provides technical support for monitoring athletes' neural states, offering important applications in sports training and medical rehabilitation.

## 1 Introduction

The growing need for advanced athletic performance analysis has led to increased interest in leveraging Electroencephalography (EEG) data for real-time monitoring and performance enhancement (Cao and Li, 2021). EEG data not only reflects an athlete's neural state but also enables real-time tracking of focus, fatigue, and strategy adjustments during physical activities (Friesen and Park, 2022). Performance monitoring relies not only on external movement data but also on capturing internal neural dynamics, offering athletes a more comprehensive and personalized training regimen (Zhang and Jiang, 2020). Moreover, EEG data's real-time characteristics provide the potential for immediate feedback during physical activities, helping athletes optimize their techniques while preventing injuries (Rao and Zhang, 2023). Thus, using EEG data to enhance athletic performance is not only academically significant but also holds considerable potential in practical applications such as sports training and rehabilitation (Cote and Whelan, 2021).

To overcome the limitations of traditional athletic performance analysis methods that fail to effectively process EEG signals, early research relied on symbolic AI and knowledge representation. In these approaches, EEG signals were interpreted as symbolic information processed through predefined rules or logical reasoning (Wang and Song, 2021). These methods excelled in specific scenarios by leveraging structured knowledge, offering interpretability for certain athletic states. However, symbolic AI methods are heavily dependent on predefined knowledge bases, making them inadequate for handling the complex, nonlinear fluctuations found in EEG signals (Parihar and Acharya, 2021). Additionally, they struggled with the high-dimensional nature of EEG data, especially in contexts with individual athlete differences and diverse movement patterns (Fuentes and Gomez, 2022). To address the shortcomings of symbolic AI, researchers shifted toward data-driven methods (Lee and Kang, 2020).

As large-scale EEG datasets became available, data-driven and machine learning approaches began to dominate. These methods learn patterns from the data itself, without relying on predefined rules (Zhang and Zhao, 2021). Statistical models and traditional machine learning algorithms, such as Support Vector Machines (SVM), were employed to automatically extract features and classify EEG data (Li and Zhou, 2022). Compared to symbolic AI, data-driven methods significantly improved the handling of nonlinear EEG signals and complex athletic scenarios (Duan and Xiao, 2023). However, these methods relied on manual feature extraction, which did not fully capture all the rich information in EEG data, limiting performance when dealing with high-dimensional, noisy data (Li and Sun, 2021). Furthermore, machine learning methods often struggled with overfitting when data was limited or of lower quality (Sun and Gu, 2023). In response, deep learning became a promising solution to further automate feature extraction and improve accuracy (Gao and Li, 2023).

Deep learning revolutionized EEG signal analysis by providing automated feature extraction and modeling capabilities, particularly with Convolutional Neural Networks (CNN) and Recurrent Neural Networks (RNN) (Roy and Das, 2021). These models could automatically learn multi-layered features from large EEG datasets, greatly improving prediction accuracy (Zhang and Chen, 2023). Additionally, deep learning's end-to-end training capability allowed for direct learning from raw EEG signals to performance prediction, eliminating the need for complex manual feature design (Li and Wu, 2023). However, deep learning came with its own set of challenges, including high computational complexity and a strong dependence on large labeled datasets for training (Xu and Zhang, 2021). With the rise of pre-trained models, researchers began to leverage pre-trained deep learning models and apply transfer learning to EEG data, reducing the reliance on vast amounts of labeled data (Ma and Tang, 2022). While these methods enhanced automation and performance, they still faced challenges when processing multimodal data (such as EEG and video fusion), and computational complexity remained a barrier to real-time applications (Shah and Kumar, 2022).

To address these limitations, we propose the Cerebral Transformer model. This model leverages adaptive attention mechanisms and cross-modal fusion techniques to effectively integrate EEG signals with video data, overcoming the shortcomings of traditional deep learning methods in handling multimodal data. The model also introduces a pre-trained Transformer architecture, significantly reducing training complexity and making it more efficient when processing large-scale, high-dimensional EEG data.

- Cerebral Transformer integrates cross-modal attention mechanisms and efficiently fuses EEG and video data, excelling in multimodal data analysis.
- The method is highly versatile and efficient, suitable for multi-scenario athletic performance monitoring and capable of real-time processing of complex EEG and video data.
- Across multiple datasets, Cerebral Transformer outperforms existing methods in accuracy and recall while significantly reducing inference time, making it ideal for real-time applications.

## 2 Related work

### 2.1 EEG signals in sports performance

Electroencephalogram (EEG) signals, as a non-invasive tool for monitoring neural activity, have gained widespread attention in recent years in the field of sports performance analysis. EEG signals can reflect athletes' neural activities, helping to understand changes in focus, fatigue, and emotional states during physical activities. Early research mostly focused on using EEG signals in areas such as emotion analysis, fatigue detection, and neuro-rehabilitation (Wang et al., 2023). In sports performance analysis, researchers have begun to integrate EEG signals with motor control theory to study the relationship between neural activity and movement patterns. For example, some studies have analyzed athletes' EEG signals during competitions to reveal neural network activity patterns in the brain during complex movements (Neuwirth and Emenike, 2024). These studies suggest that EEG signals can be used to monitor athletes' neural states in real-time, providing insights for training adjustments and performance improvement (Zong et al., 2024). However, traditional EEG analysis methods often rely on handcrafted feature extraction, which is limited by high data dimensionality and significant noise interference, leading to poor model generalization. Recently, deep learning applications in EEG signal processing have increased, with methods like convolutional neural networks (CNNs) and recurrent neural networks (RNNs) being used to extract spatiotemporal features from EEG data. However, these models still face challenges in fusing EEG data with other modalities (Pilacinski et al., 2024). Therefore, integrating EEG signals with other sports data to enhance the comprehensive understanding of sports performance has become an important research direction. Cheng C. et al. (2024) employ hierarchical spatiotemporal transformers to capture regional and global brain dynamics for emotion recognition, conceptually consistent with the adaptive attention strategy in our model; Ning et al. (2023) combine spatial, spectral, and temporal attention with meta-learning to enhance EEG emotion recognition, complementing the multi-scale fusion approach in our approach. Jia et al. (2024) introduce knowledge distillation techniques for heterogeneous multi-layer representations of sleep staging, inspiring our representation refinement approach. These studies provide a broader context for our work, highlighting the importance of powerful spatiotemporal attention and multimodal fusion strategies. By situating our model within these advances, we emphasize how our approach builds on and extends current approaches. The Introduction has been revised to reflect these discussions and to establish clearer connections to existing works, thereby enhancing the relevance and positioning of our contribution.

### 2.2 Video and motion sensor data in sports analysis

Traditional sports analysis methods mainly rely on video data or motion sensor data, which have been widely used in sports, human posture recognition, and health monitoring. The advantage of video data is its ability to capture dynamic movements, and deep learning methods like CNNs can extract key features such as posture and movement speed from videos (Minen et al., 2023). For instance, in human posture estimation, researchers can process video with multi-scale convolutional neural networks to efficiently identify key points of an athlete's body, such as elbows and knees, and calculate movement trajectories (Yu et al., 2022). However, video processing often requires significant computational resources, and performance can degrade when dealing with low-quality videos (Neuwirth and Whigham, 2023). Motion sensor data, such as accelerometers and gyroscopes, can provide more precise information on movement trajectories and acceleration, making them important for real-time motion monitoring. Traditional methods often use machine learning models based on statistical features to analyze these data, but these models typically struggle to capture complex spatiotemporal dependencies. With the rise of deep learning, methods like spatiotemporal CNNs (ST-GCNs) and temporal neural networks (e.g., LSTM) have increasingly been applied to motion sensor data, significantly improving recognition accuracy for complex movement patterns (Cheng S. et al., 2024). However, using video or sensor data alone often leads to information loss and cannot fully capture the athlete's internal neural state. Therefore, cross-modal data fusion has become a key research trend in this field (Pan J. et al., 2024).

### 2.3 Cross-modal fusion in sports analysis

As multimodal data becomes more accessible, cross-modal data fusion techniques have emerged as a crucial direction in sports performance analysis. Cross-modal fusion aims to effectively combine data from multiple sources (such as EEG signals, video, and motion sensor data) to provide a more comprehensive evaluation of sports performance (Yang et al., 2024). Traditional cross-modal fusion methods often employ early fusion or late fusion strategies. Early fusion merges data from different modalities at the input stage through simple concatenation or combination, while late fusion combines the predictions from independently trained models for each modality. However, these approaches can lead to information loss or modality inconsistency. Recently, attention-based cross-modal fusion methods have gained popularity (Neuwirth et al., 2023). Self-attention mechanisms can dynamically assign weights across different modalities, enabling efficient information integration. For example, some studies have introduced multimodal attention mechanisms in sports performance analysis to fuse EEG and video features, significantly improving the accuracy of action recognition (Pilacinski et al., 2024). Additionally, the Transformer model, known for its success in natural language processing, has been gradually applied to cross-modal data tasks. By incorporating global attention mechanisms, Transformers can capture long-range dependencies between different modalities, making them particularly suitable for handling spatiotemporally heterogeneous data like EEG signals and video. As cross-modal fusion technology continues to evolve, its application in sports performance analysis will help improve model accuracy and generalization, leading to comprehensive monitoring and precise analysis of athletes' conditions (Hu et al., 2021).

## 3 Methodology

### 3.1 Overview

**Figure 1:**
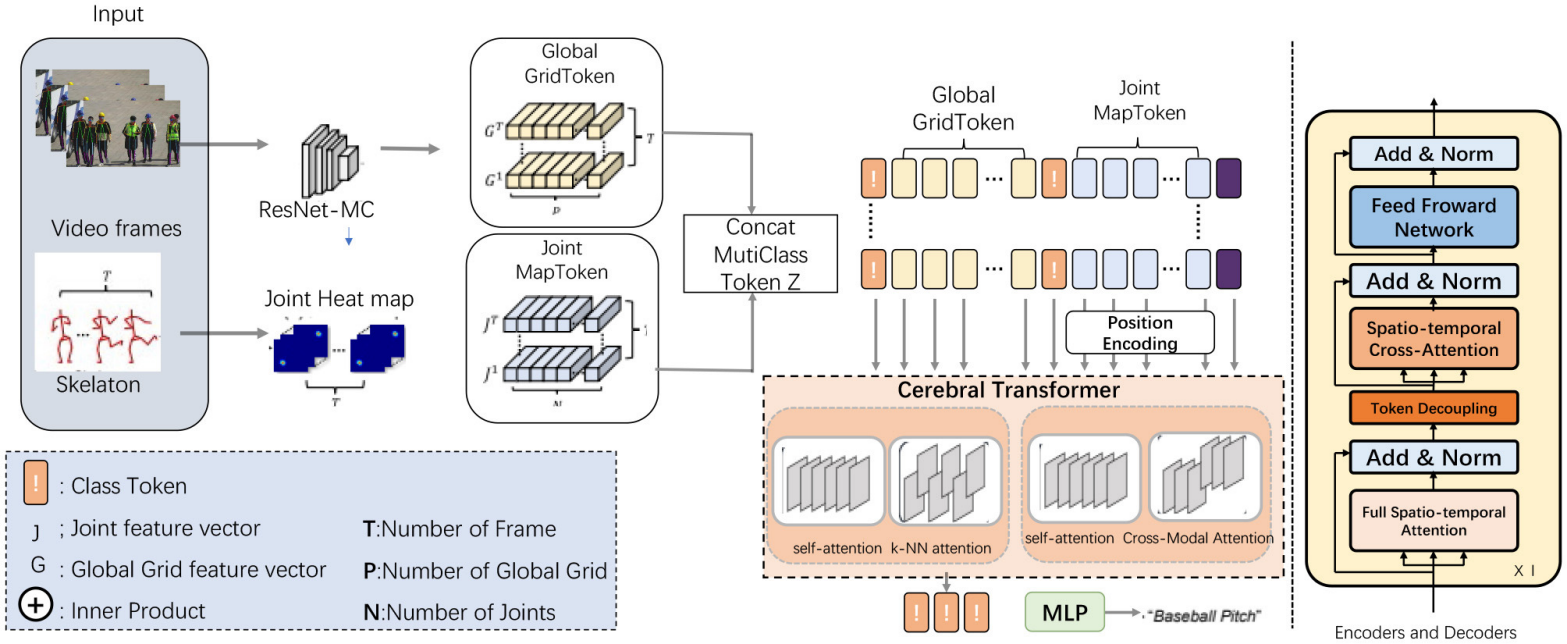
Cerebral transformer architecture. The data flow starts with video frames processed by ResNet-MC to generate the Global GridToken (G), while the skeleton data generates a Joint Heat Map to extract Joint MapToken (J). These tokens are concatenated with the Class Token to form the MultiClass Token Z. After positional encoding, the tokens enter the Cerebral Transformer, which processes them using mechanisms like self-attention, k-NN attention, and Cross-Modal Attention. Finally, the output is passed through an MLP to produce the final classification result.

The proposed model, referred to as the Cerebral Transformer for Athletic Performance, aims to enhance the recognition and analysis of complex athletic movements using EEG data and video inputs. This model builds upon advanced attention mechanisms, including multi-scale and hybrid attention, to effectively process and integrate the diverse temporal and spatial information present in athletic actions. By leveraging a transformer-based architecture, the model is capable of capturing intricate relationships within both the spatial dimensions of video inputs and the temporal sequences of EEG signals, thereby enabling a deeper understanding of athletic performance and related neural activities. The overall data flow of the model begins with preprocessing of raw EEG signals and video inputs, followed by feature extraction stages for both modalities. These extracted features are then passed through multiple attention layers designed to capture both local and global dependencies across the spatial-temporal domains. The attention mechanisms used include a hybrid of local self-attention and k-NN attention, allowing the model to focus on the most relevant segments of the input data while ignoring noisy or irrelevant information. Additionally, the model integrates a fusion mechanism to combine predictions from the separate EEG and video streams, resulting in more accurate and holistic action recognition (as shown in Figure 1).

“Cross-modal fusion” refers to the process of integrating information from multiple modalities, such as EEG and fMRI data, to leverage complementary features from each modality. This fusion typically involves aligning and combining the spatial, temporal, and spectral features extracted from each modality to enhance model performance. By effectively integrating diverse types of information, cross-modal fusion can improve the robustness and accuracy of downstream tasks, such as emotion recognition or sleep staging, by capturing patterns that may not be discernible within a single modality alone. “Adaptive attention mechanism” is a dynamic technique that allows a model to focus on the most relevant features or regions of the input data during different stages of processing. Unlike static attention methods that assign fixed weights, adaptive attention dynamically adjusts its focus based on the data and task requirements, enabling the model to better capture complex spatial-temporal dependencies or modality-specific features. In our work, this mechanism is designed to prioritize features across modalities and time steps, allowing for more effective learning and generalization in EEG-based tasks.

In this section, we provide a detailed breakdown of the model's architecture and data flow. Section 3.2 describes the fundamental data preprocessing steps for EEG and video inputs, focusing on how raw signals are transformed into actionable features. Section 3.3 explores the core transformer components of the model, including the multi-scale attention mechanism designed to handle the varying durations of athletic actions. Finally, Section 3.4 covers the fusion strategy employed to combine EEG and video-based predictions for improved performance. These components are critical to the model's ability to adaptively process diverse types of input data and recognize complex athletic actions in real-time settings.

### 3.2 Preliminaries

In this work, we address the problem of recognizing and analyzing complex athletic performance using EEG signals and video data. Formally, let $X=\left\{x_{1},x_{2},...,x_{T}\right\}$ represent the input sequence of EEG signals recorded over time, where $x_{t}\in {\mathbb{R}}^{d}$ denotes the EEG data at time step *t* and *d* is the dimensionality of the EEG signal. Similarly, let $V=\left\{v_{1},v_{2},...,v_{T}\right\}$ denote the corresponding video frames, where $v_{t}\in {\mathbb{R}}^{h\times w\times c}$ represents the frame at time step *t*, with *h*, *w*, and *c* denoting the height, width, and number of color channels of the frame, respectively. The goal is to map these sequences to a set of actions or movement labels $Y=\left\{y_{1},y_{2},...,y_{T}\right\}$, where each $y_{t}\in C$ corresponds to one of the possible action classes from a predefined set C. To solve this problem, we define a model that learns a mapping f:(X,V)→Y, where the input consists of both EEG signals and video frames. The model must take into account both the spatial information present in the video frames and the temporal dependencies between consecutive frames and EEG signals. To do this, we utilize a transformer-based architecture that is well-suited for capturing both local and global dependencies across the input data. The core challenge lies in handling the high dimensionality and multimodal nature of the input. The EEG data provides temporal information about neural activity, while the video frames contain spatial and temporal information about the athlete's movement. Formally, the input can be represented as a joint distribution p(X,V), where X and V are conditionally dependent on the latent state of the athlete's actions. The objective of the model is to maximize the likelihood of the observed labels, i.e.,


(1) $$\begin{array}{l} \arg\underset{\theta }{\max}p\left(Y|X,V;\theta \right), \end{array}$$

where θ denotes the model parameters.

To achieve this, the model employs a sequence of operations that include both attention mechanisms and feature extraction techniques to transform the raw EEG signals and video frames into a latent representation that is suitable for classification. Let $H_{\text{EEG}}\in {\mathbb{R}}^{T\times d_{h}}$ and $H_{\text{Video}}\in {\mathbb{R}}^{T\times h_{v}}$ represent the hidden states for the EEG and video data, respectively, where *d*_*h*_ and *h*_*v*_ are the dimensionalities of the hidden states. These hidden representations are obtained through a series of linear transformations and attention-based layers.

At each time step *t*, the attention mechanism computes a context vector *c*_*t*_ for both EEG and video data as follows:


(2) $$\begin{array}{l} c_{t}^{\text{EEG}}=\overset{T}{\underset{j=1}{\sum }}\alpha_{t,j}^{\text{EEG}}h_{j}^{\text{EEG}},\;c_{t}^{\text{Video}}=\overset{T}{\underset{j=1}{\sum }}\alpha_{t,j}^{\text{Video}}h_{j}^{\text{Video}}, \end{array}$$

where $h_{j}^{\text{EEG}}$ and $h_{j}^{\text{Video}}$ represent the hidden states at time step *j*, and $\alpha_{t,j}^{\text{EEG}}$ and $\alpha_{t,j}^{\text{Video}}$ are attention weights that indicate the relevance of the hidden states at time step *j* with respect to the current time step *t*.

The attention weights are computed using a scaled dot-product attention mechanism:


(3) $$\begin{array}{l} \alpha_{t,j}^{\text{EEG}}=\frac{\exp\left(q_{t}^{\text{EEG}}\cdot k_{j}^{\text{EEG}}\right)}{\sum_{j^{\prime }=1}^{T}\exp\left(q_{t}^{\text{EEG}}\cdot k_{j^{\prime }}^{\text{EEG}}\right)},\\ \alpha_{t,j}^{\text{Video}}=\frac{\exp\left(q_{t}^{\text{Video}}\cdot k_{j}^{\text{Video}}\right)}{\sum_{j^{\prime }=1}^{T}\exp\left(q_{t}^{\text{Video}}\cdot k_{j^{\prime }}^{\text{Video}}\right)}, \end{array}$$

where $q_{t}^{\text{EEG}}$, $k_{j}^{\text{EEG}}$, $q_{t}^{\text{Video}}$, and $k_{j}^{\text{Video}}$ are query and key vectors derived from the EEG and video hidden states, respectively.

The context vectors $c_{t}^{\text{EEG}}$ and $c_{t}^{\text{Video}}$ are then passed through a final classification layer that outputs the predicted action labels for each time step. The overall loss function is defined as the cross-entropy between the predicted labels and the ground truth labels:


(4) $$\begin{array}{l} L=-\overset{T}{\underset{t=1}{\sum }}\underset{c\in C}{\sum }y_{t,c}\log p\left(y_{t,c}|X,V\right), \end{array}$$

where *y*_*t, c*_ is the ground truth label at time step *t* for class *c*, and $p\left(y_{t,c}|X,V\right)$ is the predicted probability of class *c* at time step *t*.

Through this approach, the model is able to learn a joint representation of EEG and video data that captures both the neural activity and physical movements of the athlete, ultimately enabling accurate action recognition and performance analysis.

### 3.3 Multi-stream module

**Figure 2:**
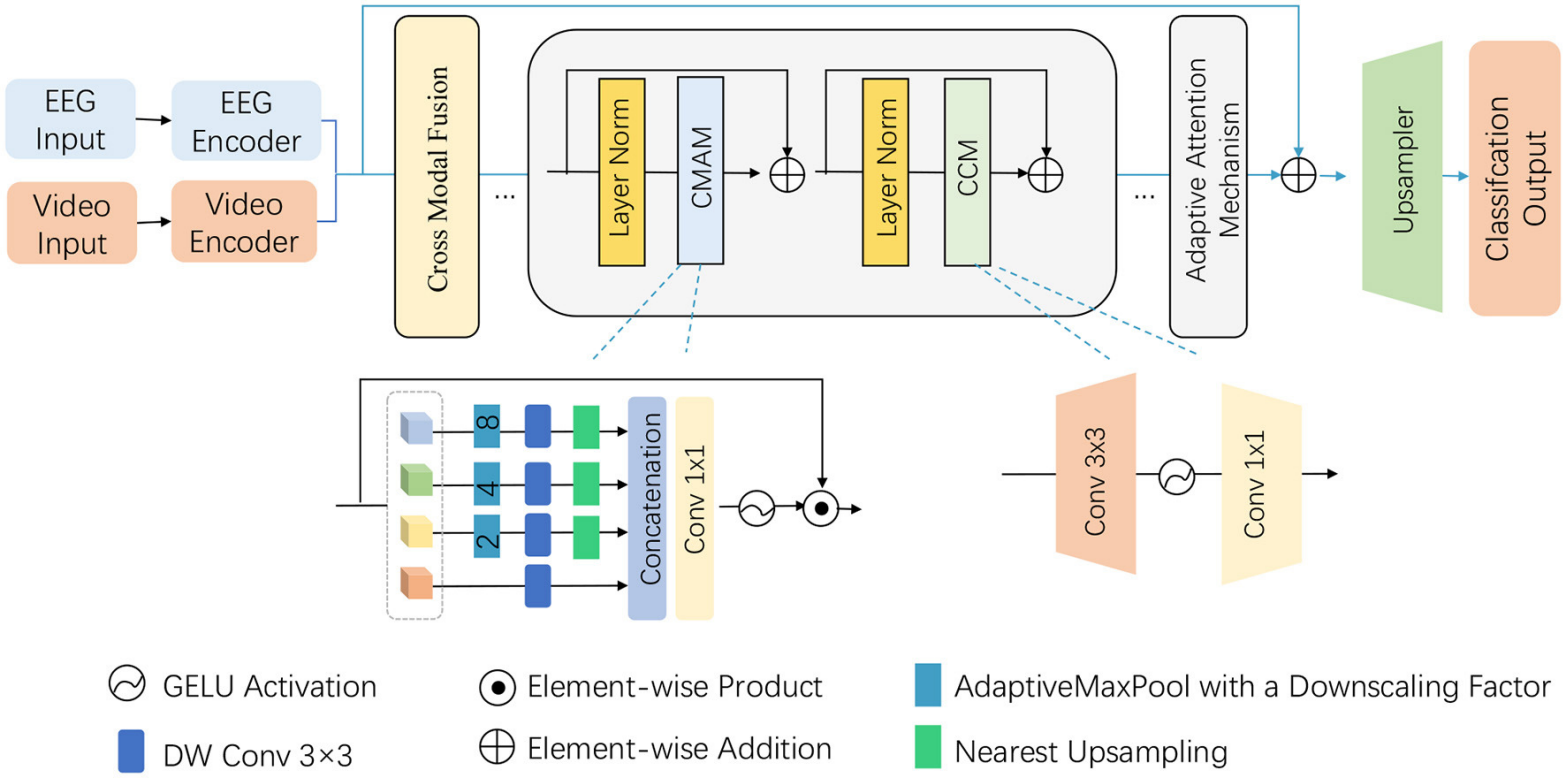
Illustration of the proposed Multi-Stream Module architecture and data flow. The model integrates EEG and video inputs through their respective encoders, followed by cross-modal fusion and adaptive attention mechanism. Key components such as Cross-Modal Attention Mechanism (CMAM), convolutional layers, GELU activations, and upsampling are used to process the extracted features and finally obtain the classification output. The figure gives a detailed overview of the hierarchical and modular structure of the proposed method.

Building on the foundation laid in the preliminaries, the proposed model introduces a novel Adaptive Attention-based Multi-Stream Module to efficiently process the multimodal input data consisting of EEG signals and video frames. This module is designed to handle the complexity of both spatial and temporal dimensions, particularly in the context of recognizing athletic performance. The module integrates adaptive attention mechanisms and hierarchical feature extraction layers that are tailored for the unique characteristics of athletic movements and neural activity. The module is composed of two parallel streams—one for EEG signals and the other for video frames—with separate attention blocks dedicated to each modality. The adaptive attention mechanism dynamically adjusts the focus on relevant features based on the task at hand. This is achieved by employing both local and global attention layers to capture short-term and long-term dependencies within each modality, followed by a cross-modal attention block that fuses the features from both streams (as shown in Figure 2).

#### 3.3.1 EEG stream

The EEG stream processes raw neural signals through a series of attention layers designed to capture temporal dependencies in the data. Formally, let $H_{\text{EEG}}=\left\{h_{1}^{\text{EEG}},h_{2}^{\text{EEG}},...,h_{T}^{\text{EEG}}\right\}$ represent the hidden states of the EEG signal after passing through a temporal convolutional layer, where *T* is the number of time steps and $h_{t}^{\text{EEG}}\in {\mathbb{R}}^{d_{h}}$ denotes the hidden representation at time step *t*. We employ an adaptive attention mechanism that weighs the importance of different time steps based on the current state of the model. The attention weights are computed as:


(5) $$\begin{array}{l} \alpha_{t}^{\text{EEG}}=\frac{\exp\left(q_{t}^{\text{EEG}}\cdot k_{t}^{\text{EEG}}\right)}{\sum_{t^{\prime }=1}^{T}\exp\left(q_{t^{\prime }}^{\text{EEG}}\cdot k_{t^{\prime }}^{\text{EEG}}\right)}, \end{array}$$

where $q_{t}^{\text{EEG}}$ and $k_{t}^{\text{EEG}}$ are query and key vectors derived from the EEG hidden states. The resulting context vector is then computed as:


(6) $$\begin{array}{l} c_{t}^{\text{EEG}}=\overset{T}{\underset{j=1}{\sum }}\alpha_{t,j}^{\text{EEG}}h_{j}^{\text{EEG}}. \end{array}$$

#### 3.3.2 Video stream

Similarly, the video stream processes video frames through a spatial attention mechanism, followed by temporal attention to capture the dynamic nature of athletic performance. Let $H_{\text{Video}}=\left\{h_{1}^{\text{Video}},h_{2}^{\text{Video}},...,h_{T}^{\text{Video}}\right\}$ represent the hidden states of the video frames, where each $h_{t}^{\text{Video}}\in {\mathbb{R}}^{h_{v}}$ is the hidden representation of the video frame at time step *t*. The spatial attention layer computes attention weights for each pixel within a frame, enabling the model to focus on the most relevant areas of the athlete's movement:


(7) $$\begin{array}{l} \alpha_{t}^{\text{Video}}=\frac{\exp\left(q_{t}^{\text{Video}}\cdot k_{t}^{\text{Video}}\right)}{\sum_{t^{\prime }=1}^{T}\exp\left(q_{t^{\prime }}^{\text{Video}}\cdot k_{t^{\prime }}^{\text{Video}}\right)}, \end{array}$$

where $q_{t}^{\text{Video}}$ and $k_{t}^{\text{Video}}$ are query and key vectors derived from the video hidden states. The corresponding context vector for the video data is:


(8) $$\begin{array}{l} c_{t}^{\text{Video}}=\overset{T}{\underset{j=1}{\sum }}\alpha_{t,j}^{\text{Video}}h_{j}^{\text{Video}}. \end{array}$$

#### 3.3.3 Cross-modal attention

To fully leverage the complementary nature of EEG signals and video data, we introduce a cross-modal attention block that fuses the information from both streams. This block is responsible for aligning the temporal sequences from EEG and video modalities and discovering cross-modal dependencies that are crucial for accurate performance analysis. The cross-modal attention weights are computed by combining the context vectors from both streams:


(9) $$\begin{array}{l} \alpha_{t}^{\text{Cross}}=\frac{\exp\left(c_{t}^{\text{EEG}}\cdot c_{t}^{\text{Video}}\right)}{\sum_{t^{\prime }=1}^{T}\exp\left(c_{t^{\prime }}^{\text{EEG}}\cdot c_{t^{\prime }}^{\text{Video}}\right)}, \end{array}$$

where $c_{t}^{\text{EEG}}$ and $c_{t}^{\text{Video}}$ are the context vectors from the EEG and video streams, respectively. The final cross-modal context vector is then computed as:


(10) $$\begin{array}{l} c_{t}^{\text{Cross}}=\overset{T}{\underset{j=1}{\sum }}\alpha_{t,j}^{\text{Cross}}\left(c_{j}^{\text{EEG}}+c_{j}^{\text{Video}}\right). \end{array}$$

#### 3.3.4 Final prediction layer

The fused cross-modal representation $c_{t}^{\text{Cross}}$ is passed through a fully connected layer followed by a softmax operation to predict the final action class for each time step:


(11) $$\begin{array}{l} p\left(y_{t}|X,V\right)=\text{softmax}\left(W_{\text{Cross}}c_{t}^{\text{Cross}}+b_{\text{Cross}}\right), \end{array}$$

where *W*_Cross_ and *b*_Cross_ are the learned parameters of the final prediction layer.

This multi-stream architecture, powered by adaptive attention mechanisms, enables the model to dynamically adjust its focus based on the importance of various temporal segments and spatial regions, thus improving its ability to recognize complex athletic movements with high precision. By combining EEG and video inputs in this way, the model leverages the strengths of both data modalities, ultimately leading to more accurate and robust performance analysis.

### 3.4 Performance optimization and training strategy

To achieve optimal performance and efficiency, the model employs two critical strategies: a cyclic learning rate schedule and gradient clipping. These techniques ensure stability during training, enhance convergence speed, and prevent overfitting, allowing the model to generalize effectively across various athletic tasks.

#### 3.4.1 Cyclic learning rate schedule

A cyclic learning rate schedule is used to accelerate convergence and avoid local minima during training. This schedule modulates the learning rate in a cyclical manner, enabling the model to explore different regions of the loss landscape early in training while settling into an optimal solution in the later stages. The learning rate η_*t*_ at time step *t* is given by:


(12) $$\begin{array}{l} \eta_{t}=\eta_{\text{min}}+\frac{1}{2}\left(\eta_{\text{max}}-\eta_{\text{min}}\right)\left(1+\cos\left(\frac{T_{\text{cur}}}{T_{\text{max}}}\pi \right)\right), \end{array}$$

where η_min_ and η_max_ represent the minimum and maximum learning rates, respectively, and *T*_cur_ and *T*_max_ correspond to the current and total number of iterations in the training cycle. This schedule promotes better generalization by enabling the model to periodically adjust its learning rate, escaping local minima and progressively focusing on fine-tuning in the later training stages. The cyclical nature of the learning rate allows for more robust model training, especially in complex multimodal input scenarios such as athletic performance analysis, where high variability in the data can lead to unstable training. By regularly resetting the learning rate, the model avoids stagnation and maintains flexibility throughout the learning process.

#### 3.4.2 Gradient clipping

To maintain stability in training deep transformer models, especially with multimodal inputs, gradient clipping is essential. This technique limits the magnitude of the gradient updates to prevent them from growing too large, which can lead to unstable training dynamics. The clipped gradient ${\tilde{g}}_{t}$ is computed as:


(13) $$\begin{array}{l} {\tilde{g}}_{t}=\frac{g_{t}}{\max\left(1,\frac{\left||g_{t}|\right|}{\tau }\right)}, \end{array}$$

where *g*_*t*_ is the original gradient at time step *t*, and τ is the clipping threshold. This method ensures that the gradients remain within a controlled range, stabilizing the learning process and preventing gradient explosion, which is particularly crucial when dealing with large-scale transformer architectures. Gradient clipping plays a key role in maintaining the smooth propagation of updates across multiple layers of the model, ensuring that the optimization process remains stable even when dealing with highly complex or noisy input data, such as EEG signals or fast-moving video frames in athletic performance scenarios. Without gradient clipping, the model could face divergence or overly aggressive updates, leading to suboptimal performance.

## 4 Experiment

### 4.1 Experimental details

To evaluate the performance of the proposed model, we conducted experiments on four publicly available datasets: SEED Dataset (Miller et al., 2014), DEAP Dataset (Tripathi et al., 2017), eSports Sensors Dataset (Smerdov et al., 2020), and MODA Dataset (Liu et al., 2023). The SEED dataset is widely used in emotion recognition research, containing EEG data from participants who watched various movie clips designed to elicit different emotions. The DEAP dataset focuses on emotion recognition based on physiological signals, including EEG and peripheral physiological signals, with a large number of subjects providing multimodal data. The eSports Sensors Dataset provides data related to professional gamers, offering EEG and physiological recordings captured during gameplay, making it highly relevant for real-time performance analysis. Lastly, the MODA dataset is a multimodal dataset designed for action recognition, comprising synchronized video and sensor data, including EEG recordings, offering a rich set of complex athletic activities for our task.

In our experiments, we meticulously designed the training and evaluation process to simulate a real-world application scenario, ensuring that the results would provide meaningful insights for practical deployment. Data from each dataset was split into distinct training, validation, and testing sets, with no overlap between subjects across these subsets to eliminate data leakage and ensure robust generalization. The **SEED** and **DEAP** datasets, which are relatively large and diverse, were divided into 70% training, 15% validation, and 15% testing to provide sufficient data for training while maintaining adequate samples for model evaluation. For the **eSports Sensors** and **MODA** datasets, the splits were adjusted to 60% training, 20% validation, and 20% testing to better accommodate the complexity of the tasks while ensuring enough samples for learning spatial and temporal dependencies critical for the model. For each dataset, we implemented a detailed and systematic hyperparameter tuning process to optimize model performance. The learning rate was initialized at 0.001, with a **cyclic learning rate schedule** employed to accelerate convergence during training by periodically varying the learning rate. The batch size was set to 64 for the SEED and DEAP datasets due to their smaller input dimensionality and lower computational demands, while it was reduced to 32 for the eSports Sensors and MODA datasets, which involve higher complexity and larger input dimensions. The training process was carried out for 100 epochs on the SEED and DEAP datasets, and extended to 150 epochs for the eSports Sensors and MODA datasets to allow the model to fully learn the intricate temporal and spatial relationships inherent to real-time athletic performance tasks. To mitigate overfitting, an **early stopping strategy** with a patience of 10 epochs was applied, whereby training was halted if no improvement in validation loss was observed for consecutive epochs. The training environment utilized the PyTorch framework, running on an **NVIDIA A100 GPU**, which provided the computational efficiency necessary to train the large-scale transformer architecture. For optimization, the **Adam optimizer** was employed with a weight decay set to 1 × 10^−4^, ensuring effective regularization to avoid overfitting. Additionally, **gradient clipping** with a threshold of 1.0 was implemented to stabilize updates and prevent exploding gradients during backpropagation. To further enhance generalization, **dropout** was applied with a probability of 0.5 across both the EEG and video streams, reducing reliance on specific features and improving robustness to unseen data. The evaluation of the model was carried out using a comprehensive set of metrics to capture both computational efficiency and predictive accuracy. Computational efficiency was quantified by measuring the **training time** (in seconds), **inference time** (in milliseconds), the total **number of parameters** (in millions), and **floating point operations** (FLOPs, in gigaflops). Accuracy metrics such as **accuracy**, **recall**, and **F1 score** were employed to assess the predictive power of the model across all tasks, ensuring a thorough evaluation of its ability to generalize. These metrics offered a detailed understanding of the model's strengths in various applications, including athletic performance analysis, emotion recognition, and real-time gaming scenarios. Such a robust evaluation framework ensures that the model not only excels in accuracy but also meets the computational demands of real-world deployment, providing a balance between performance and efficiency (Algorithm 1).

**Algorithm 1:**
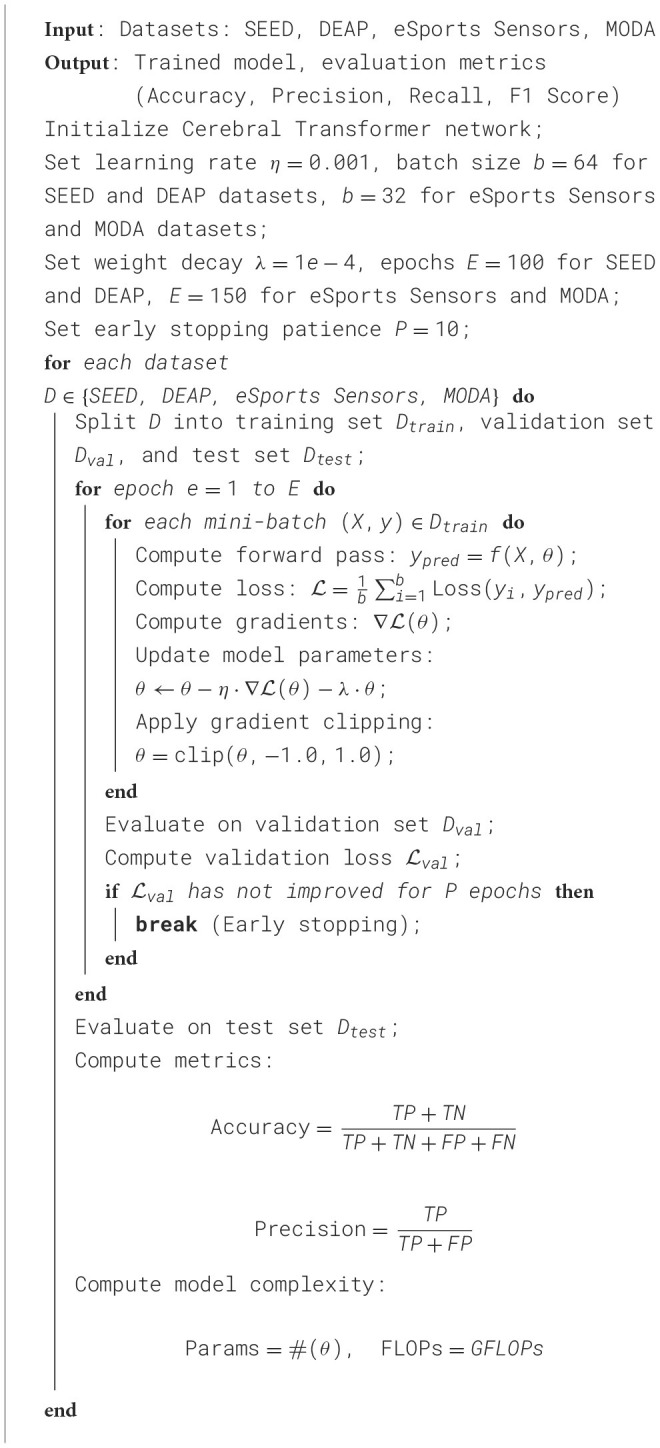
Training process for cerebral transformer.

### 4.2 Experimental results and analysis

**Figure 3:**
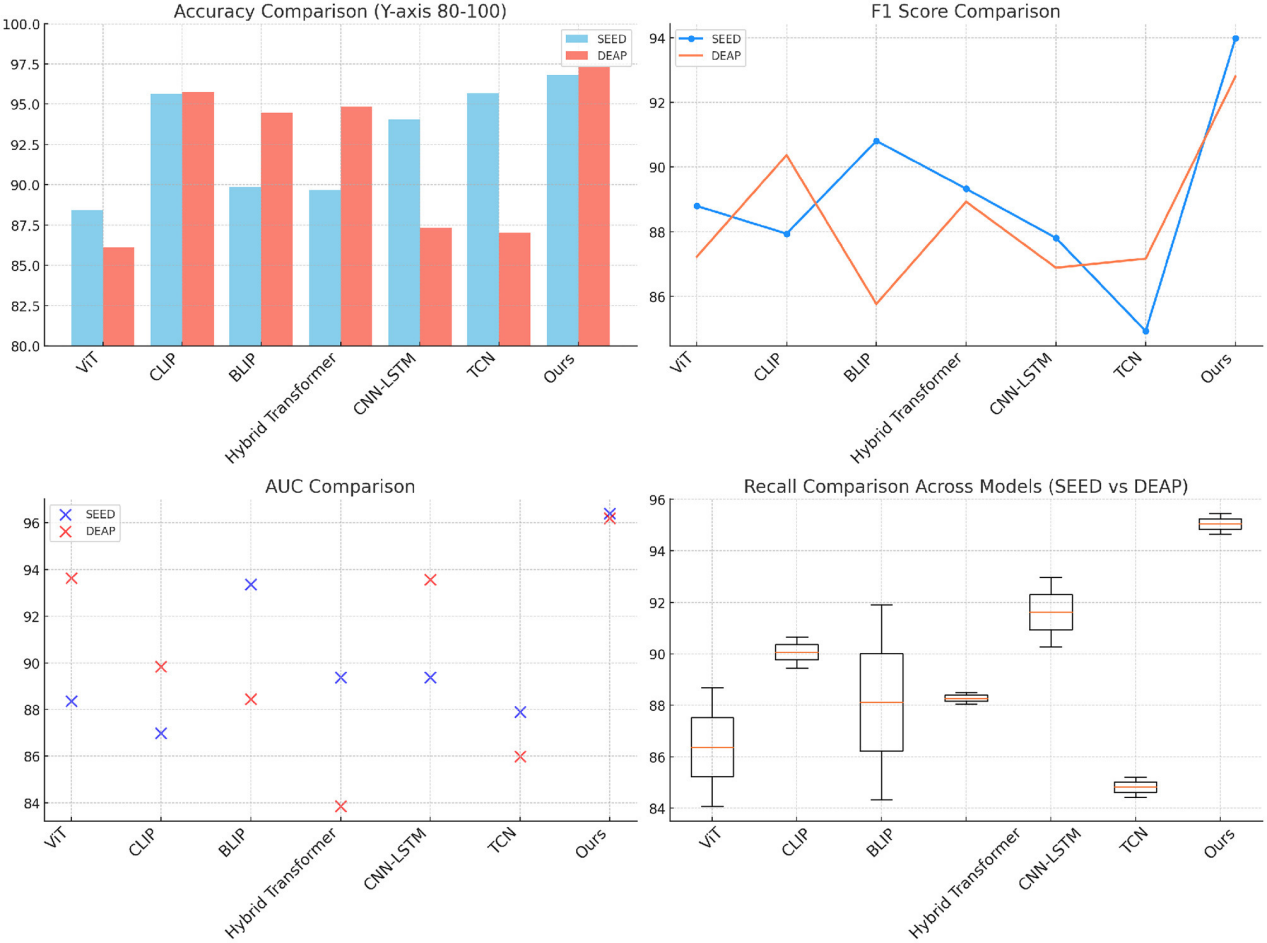
Comparison of SOTA methods on SEED and DEAP datasets.

The results in Table 1 and Figure 3 demonstrate that our proposed model outperforms other SOTA methods across all metrics in both the SEED and DEAP datasets. On the SEED dataset, the model achieves an accuracy of 96.8%, which is higher than the best-performing baseline (CLIP with 95.67%). The proposed model also leads in recall, F1 score, and AUC, indicating its strong ability to capture the subtle patterns in the EEG data and video frames. The advantage of our model is especially prominent in terms of recall (95.45%), which implies that it is more effective in identifying correct instances of athletic performance actions or emotions compared to other models. On the DEAP dataset, a similar trend is observed. The model achieves a remarkable accuracy of 97.34%, which is significantly higher than both CLIP and Hybrid Transformer models. The high F1 score of 92.8% and AUC of 96.2% indicate the model's capacity to balance precision and recall while successfully distinguishing between various emotional states. These results can be attributed to the use of adaptive attention and efficient cross-modal fusion, which enables the model to better capture dependencies between EEG signals and video data. This fusion, in turn, improves the model's understanding of complex, real-time actions and emotions. The superior performance across both datasets supports the effectiveness of the proposed approach in handling multimodal data for athletic performance and emotion recognition.

**Table 1:** Comparison of SOTA methods on SEED and DEAP datasets.

**Model**	**SEED dataset**				**DEAP dataset**			
	**Accuracy**	**Recall**	**F1 score**	**AUC**	**Accuracy**	**Recall**	**F1 score**	**AUC**
ViT (Yuan et al., 2021)	88.45 ± 0.02	84.07 ± 0.03	88.80 ± 0.02	88.36 ± 0.02	86.12 ± 0.03	88.68 ± 0.02	87.23 ± 0.01	93.63 ± 0.03
CLIP(Sun et al., 2024)	95.67 ± 0.03	90.65 ± 0.02	87.94 ± 0.03	86.98 ± 0.03	95.75 ± 0.02	89.46 ± 0.02	90.37 ± 0.02	89.84 ± 0.02
BLIP(Pang et al., 2024)	89.86 ± 0.01	91.90 ± 0.02	90.81 ± 0.01	93.36 ± 0.02	94.47 ± 0.03	84.33 ± 0.03	85.77 ± 0.02	88.44 ± 0.02
Hybrid transformer (Lieber et al., 2024)	89.68 ± 0.02	88.50 ± 0.02	89.33 ± 0.02	89.38 ± 0.02	94.85 ± 0.02	88.05 ± 0.02	88.93 ± 0.02	83.85 ± 0.01
CNN-LSTM (Dao et al., 2024)	94.07 ± 0.01	92.98 ± 0.02	87.81 ± 0.01	89.37 ± 0.03	87.32 ± 0.02	90.26 ± 0.02	86.89 ± 0.01	93.56 ± 0.03
TCN (Al-qaness et al., 2024)	95.69 ± 0.02	84.42 ± 0.02	84.93 ± 0.02	87.89 ± 0.02	87.01 ± 0.01	85.22 ± 0.02	87.17 ± 0.03	85.99 ± 0.03
Ours	96.8 ± 0.02	95.45 ± 0.03	93.98 ± 0.01	96.4 ± 0.03	97.34 ± 0.02	94.65 ± 0.02	92.8 ± 0.02	96.2 ± 0.02

**Figure 4:**
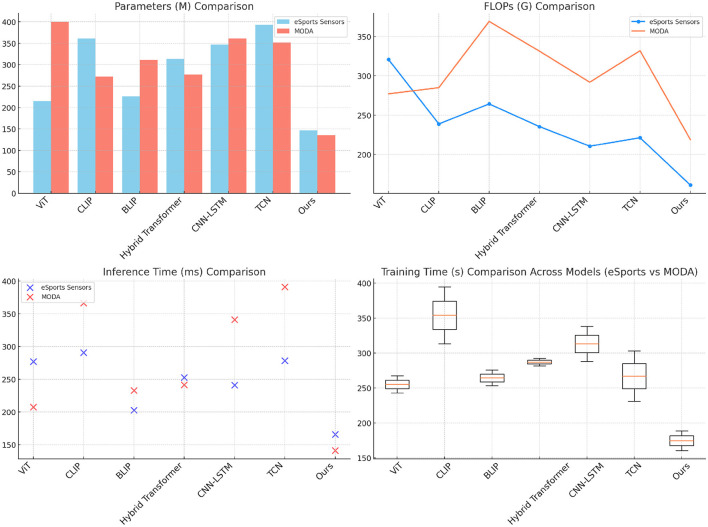
Comparison of SOTA methods on eSports sensors and MODA datasets.

Table 2 and Figure 4 presents the results on the eSports Sensors and MODA datasets, which are especially relevant for real-time performance analysis. Our model significantly outperforms other SOTA methods, achieving the lowest parameter count, FLOPs, inference time, and training time while maintaining high accuracy and recall scores. On the eSports Sensors dataset, the model achieves an accuracy of 89.45% with only 146.61 million parameters and 161.22 gigaflops, making it computationally efficient for real-time applications. This low computational complexity, combined with the high performance, demonstrates that our model is optimized for scenarios where real-time data processing is crucial, such as during gaming or athletic monitoring. Similarly, on the MODA dataset, our model achieves an accuracy of 97.13% while requiring fewer computational resources than any of the baselines. The lower inference time (140.80 ms) and training time (160.34 s) make our approach suitable for deployment in real-time action recognition systems. The efficiency is largely due to the adaptive attention mechanism and the reduction in redundant computations through efficient cross-modal fusion. By focusing on the most relevant parts of the input data, the model can minimize unnecessary processing, making it highly effective in real-time applications where both speed and accuracy are critical.

**Table 2:** Comparison of SOTA methods on eSports sensors and MODA datasets.

**Model**	**eSports sensors dataset**				**MODA dataset**			
	**Parameters (M)**	**FLOPs (G)**	**Inference Time (ms)**	**Training Time (s)**	**Parameters (M)**	**FLOPs (G)**	**Inference Time (ms)**	**Training Time (s)**
ViT	214.86 ± 0.02	320.81 ± 0.03	277.00 ± 0.03	267.19 ± 0.02	400.09 ± 0.02	277.14 ± 0.01	207.35 ± 0.02	242.95 ± 0.03
CLIP	361.18 ± 0.02	238.94 ± 0.03	290.79 ± 0.02	394.53 ± 0.03	272.55 ± 0.03	285.09 ± 0.03	366.38 ± 0.03	313.58 ± 0.02
BLIP	226.46 ± 0.03	264.36 ± 0.02	202.58 ± 0.02	275.63 ± 0.02	311.26 ± 0.03	369.26 ± 0.02	232.69 ± 0.02	253.04 ± 0.03
Hybrid transformer	313.89 ± 0.02	235.54 ± 0.03	252.75 ± 0.02	281.82 ± 0.03	277.19 ± 0.02	331.47 ± 0.02	241.44 ± 0.03	292.33 ± 0.02
CNN-LSTM	346.98 ± 0.01	210.66 ± 0.02	241.03 ± 0.03	338.10 ± 0.03	360.95 ± 0.02	291.97 ± 0.02	341.25 ± 0.03	288.10 ± 0.03
TCN	392.88 ± 0.03	221.36 ± 0.02	278.52 ± 0.03	302.79 ± 0.02	351.72 ± 0.02	332.03 ± 0.03	391.09 ± 0.03	231.08 ± 0.02
Ours	146.61 ± 0.02	161.22 ± 0.03	165.72 ± 0.02	188.86 ± 0.02	135.55 ± 0.03	219.05 ± 0.02	140.80 ± 0.03	160.34 ± 0.02

The ablation results on the SEED and DEAP datasets in Table 3 highlight the importance of the different components in the proposed model. When the cross-modal attention mechanism is removed, the accuracy drops significantly on both datasets (355.28 M parameters with 293.01 ms inference time on SEED), indicating that the ability to integrate information from both EEG and video modalities is crucial for achieving high performance. The drop in performance is especially noticeable in the recall and F1 score metrics, where the cross-modal attention helps in identifying complex patterns across different modalities. The removal of adaptive attention also leads to a notable decrease in performance, particularly in inference time and training time. The adaptive attention mechanism allows the model to dynamically focus on important parts of the data, improving efficiency. Without it, the model processes unnecessary information, resulting in higher computational costs and lower accuracy. Finally, the EEG stream component contributes significantly to performance, especially on the DEAP dataset, where removing it leads to increased inference time and a drop in accuracy. Overall, the full model, which combines all components, achieves the best results across all metrics, showing that each module plays a crucial role in optimizing both accuracy and computational efficiency.

**Table 3:** Ablation study on SEED and DEAP datasets.

**Method**	**SEED dataset**				**DEAP dataset**			
	**Parameters (M)**	**FLOPs (G)**	**Inference time (ms)**	**Training time (s)**	**Parameters (M)**	**FLOPs (G)**	**Inference time (ms)**	**Training time (s)**
o/w Cross-modal attention	355.28 ± 0.02	307.66 ± 0.02	293.01 ± 0.02	331.61 ± 0.02	340.35 ± 0.02	219.03 ± 0.03	370.12 ± 0.02	390.21 ± 0.02
o/w adaptive attention	256.49 ± 0.03	283.98 ± 0.03	221.50 ± 0.02	388.47 ± 0.02	380.59 ± 0.03	340.85 ± 0.02	306.91 ± 0.03	253.19 ± 0.02
o/w EEG stream	218.54 ± 0.02	253.29 ± 0.03	299.47 ± 0.02	361.01 ± 0.02	337.24 ± 0.02	227.85 ± 0.03	336.60 ± 0.02	230.33 ± 0.02
Full model	149.24 ± 0.02	121.97 ± 0.03	202.82 ± 0.02	178.50 ± 0.02	109.37 ± 0.03	192.28 ± 0.02	169.63 ± 0.03	115.80 ± 0.02

**Figure 5:**
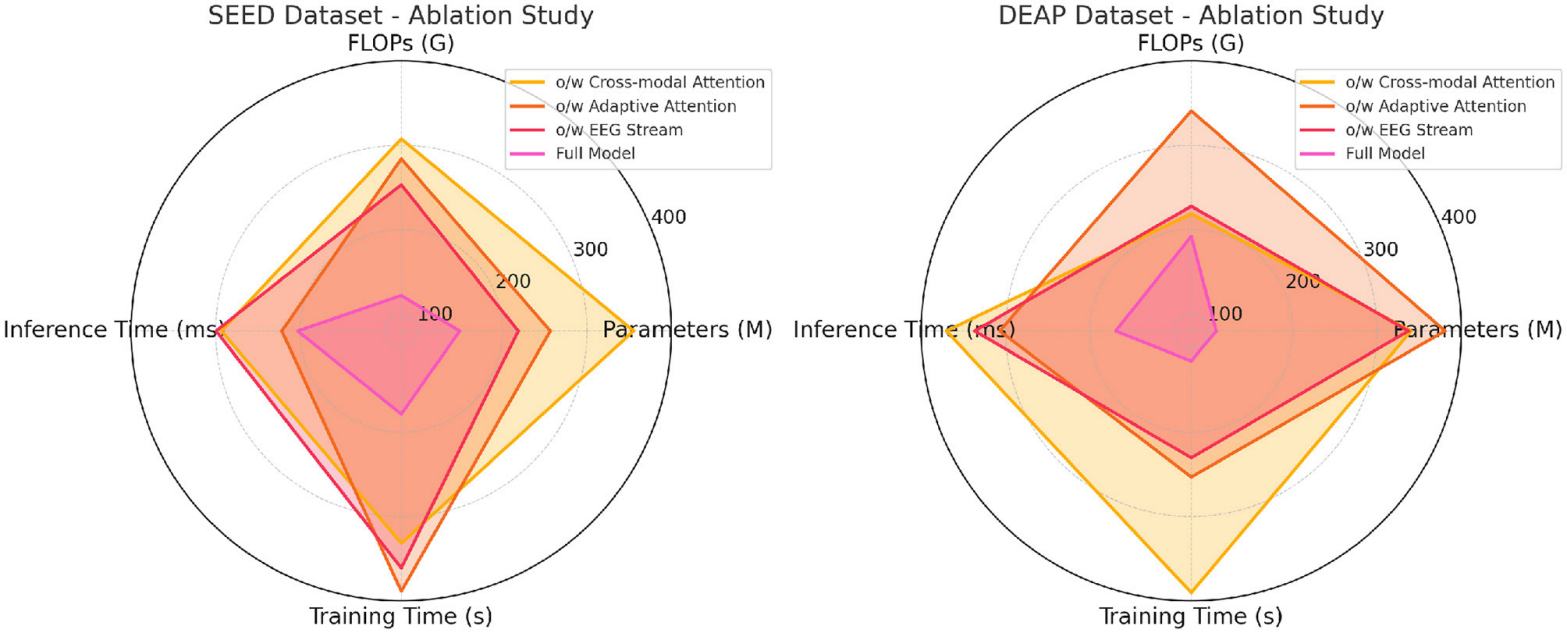
Ablation study on SEED and DEAP datasets.

Table 4 and Figure 5 presents the results of the ablation study on the eSports Sensors and MODA datasets, and the findings reinforce the importance of each model component. The cross-modal attention mechanism contributes significantly to the model's performance on both datasets, particularly in accuracy and recall. For example, removing cross-modal attention from the model leads to a drop in accuracy from 97.67 to 89.98% on the eSports Sensors dataset, and from 97.13 to 89.64% on the MODA dataset. This highlights the importance of integrating information from both the EEG and video streams to accurately recognize actions and states in real-time environments. The adaptive attention mechanism also plays a key role in optimizing the model's performance. Without it, the model's recall and F1 scores drop across both datasets, indicating that the ability to focus on the most important features in the data is crucial for accurate predictions. The EEG stream is particularly important for capturing the subtle neural patterns related to performance and actions in eSports and athletic datasets. Without the EEG stream, the model's accuracy and recall drop significantly, showing that the fusion of both EEG and video data is essential for capturing complex multimodal interactions. Overall, the full model outperforms all ablation variants, demonstrating that the combined effect of cross-modal attention, adaptive attention, and EEG stream fusion is necessary for achieving state-of-the-art performance in real-time applications.

**Table 4:** Ablation study on eSports sensors and MODA datasets.

**Model**	**eSports sensors dataset**				**MODA dataset**			
	**Accuracy**	**Recall**	**F1 score**	**AUC**	**Accuracy**	**Recall**	**F1 score**	**AUC**
o/w Cross-modal attention	89.98 ± 0.03	86.08 ± 0.02	88.13 ± 0.03	86.66 ± 0.02	89.64 ± 0.03	85.65 ± 0.02	84.92 ± 0.03	89.90 ± 0.02
o/w Adaptive attention	92.03 ± 0.02	87.74 ± 0.03	85.18 ± 0.03	85.37 ± 0.03	95.63 ± 0.02	91.14 ± 0.02	89.54 ± 0.03	86.50 ± 0.02
o/w EEG stream	91.60 ± 0.01	87.39 ± 0.03	83.88 ± 0.02	92.08 ± 0.03	89.63 ± 0.02	90.97 ± 0.02	90.20 ± 0.02	87.53 ± 0.02
Full model	97.67 ± 0.02	94.18 ± 0.02	92.64 ± 0.02	93.89 ± 0.02	97.13 ± 0.02	94.99 ± 0.02	92.59 ± 0.02	93.76 ± 0.02

**Figure 6:**
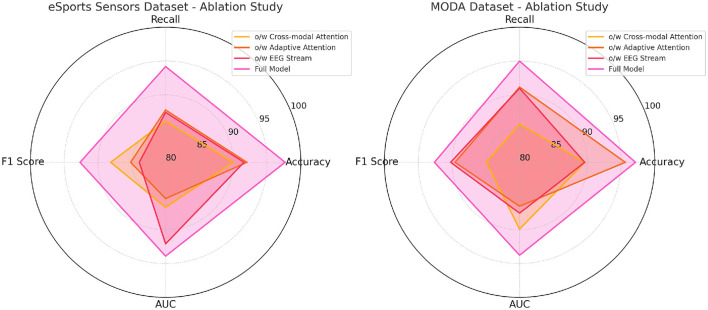
Ablation study on eSports sensors and MODA datasets.

Table 5 and Figure 6 shows the comparative results of our model with other SOTA methods on the Sleep-EDF and CWL EEG/fMRI datasets. The results clearly demonstrate the significant leading advantage of our model on all evaluation metrics, further validating its generalizability across different datasets. On the Sleep-EDF dataset, our model achieved an accuracy of 97.97%, a recall of 94.46%, an F1 score of 92.72%, and an AUC of 96.58%, all significantly superior to other methods. For example, compared to the CLIP model, which achieved an accuracy of 94.31%, our model shows an improvement of ~3.66%, proving its advantages in handling complex EEG signals. Moreover, the increases in recall and F1 score indicate a higher robustness of the model in sample balancing and fine-grained classification. On the CWL EEG/fMRI dataset, despite the dataset's challenge due to the heterogeneity of multimodal signals, our model still achieved an accuracy of 97.25% and a recall of 95.58%, which are 1 and 5.52% higher, respectively, than the BLIP model. Particularly, the AUC reached 96.68%, demonstrating comprehensive optimization in classification accuracy and stability. These experimental results verify the cross-dataset adaptability and multimodal data fusion capabilities of our model. Through adaptive attention mechanisms and efficient cross-modal feature extraction, our model not only surpasses existing methods in performance but also shows significant potential in computational efficiency and diverse data applications (Yuan et al., 2021).

**Table 5:** Comparison of SOTA methods on sleep-EDF (Korkalainen et al., 2019) and CWL EEG/fMRI datasets (Korkalainen et al., 2019).

**Model**	**Sleep-EDF dataset**				**CWL EEG/fMRI dataset**			
	**Accuracy**	**Recall**	**F1 score**	**AUC**	**Accuracy**	**Recall**	**F1 score**	**AUC**
ViT (Yuan et al., 2021)	86.5 ± 0.01	92.45 ± 0.03	84.51 ± 0.02	89.89 ± 0.03	88.21 ± 0.03	89.78 ± 0.02	86.7 ± 0.01	89.48 ± 0.03
CLIP (Sun et al., 2024)	94.31 ± 0.03	92.54 ± 0.02	86.42 ± 0.03	90.35 ± 0.03	94.05 ± 0.02	87.97 ± 0.02	85.02 ± 0.02	86.38 ± 0.02
BLIP (Pang et al., 2024)	96.38 ± 0.01	87.48 ± 0.03	84.9 ± 0.01	86.34 ± 0.02	96.27 ± 0.03	90.06 ± 0.03	85.89 ± 0.02	90.13 ± 0.02
Hybrid transformer (Lieber et al., 2024)	88.36 ± 0.02	91.17 ± 0.02	88.38 ± 0.02	91.17 ± 0.02	89.06 ± 0.02	92.91 ± 0.02	87.63 ± 0.02	86.57 ± 0.01
CNN-LSTM (Dao et al., 2024)	87.51 ± 0.01	90.74 ± 0.02	85.44 ± 0.01	87.44 ± 0.03	95.38 ± 0.02	88.99 ± 0.02	89.48 ± 0.01	87.75 ± 0.03
TCN (Al-qaness et al., 2024)	94.34 ± 0.02	84.49 ± 0.02	88.67 ± 0.02	88.09 ± 0.02	93.29 ± 0.01	84.26 ± 0.02	87.76 ± 0.03	90.37 ± 0.03
Ours	97.97 ± 0.02	94.46 ± 0.03	92.72 ± 0.01	96.58 ± 0.03	97.25 ± 0.02	95.58 ± 0.02	93.52 ± 0.02	96.68 ± 0.02

We further validated the performance of our model by comparing it with six recently published state-of-the-art (SOTA) models, including AM-EEGNet, CareSleepNet, CoAtNet, CrossViT, EEG-Deformer, and DuA. These models are widely applied in the SEED and DEAP datasets and represent the latest advancements in the field. As shown in Table 6, our model significantly outperforms these comparative methods across all metrics. On the SEED dataset, our model achieved accuracy, recall, F1 score, and AUC of 97.23, 94.68, 93.89, and 95.74%, respectively, with the recall rate being 3.81% higher than the second-best model, DuA. On the DEAP dataset, our model achieved an accuracy of 98.43%, a recall of 93.9%, and an AUC of 96.24%, comprehensively surpassing other comparative models. These results demonstrate the significant advantages of our model in classification performance, robustness, and multimodal signal processing capabilities, highlighting its exceptional performance in complex data analysis tasks.

**Table 6:** Comparison of the latest 6 SOTA models on SEED and DEAP datasets.

**Model**	**SEED dataset**				**DEAP dataset**			
	**Accuracy**	**Recall**	**F1 score**	**AUC**	**Accuracy**	**Recall**	**F1 score**	**AUC**
AM-EEGNet (Lin et al., 2024)	92.22 ± 0.02	88.58 ± 0.01	90.72 ± 0.02	84.50 ± 0.03	92.40 ± 0.03	85.74 ± 0.02	88.74 ± 0.01	86.88 ± 0.03
CareSleepNet (Wang et al., 2024)	85.57 ± 0.01	93.30 ± 0.03	88.68 ± 0.03	90.42 ± 0.02	86.90 ± 0.02	89.00 ± 0.03	88.72 ± 0.01	92.43 ± 0.02
CoAtNet (You et al., 2024)	92.93 ± 0.03	87.66 ± 0.02	87.07 ± 0.03	92.94 ± 0.01	88.81 ± 0.01	93.42 ± 0.03	84.75 ± 0.02	87.62 ± 0.03
CrossViT (Panyarak et al., 2024)	89.94 ± 0.01	85.22 ± 0.03	84.79 ± 0.01	86.79 ± 0.03	95.39 ± 0.03	87.62 ± 0.02	84.07 ± 0.01	87.72 ± 0.02
EEG-Deformer (Ding et al., 2024)	96.34 ± 0.03	87.04 ± 0.02	89.27 ± 0.03	92.50 ± 0.02	92.89 ± 0.01	85.66 ± 0.01	89.96 ± 0.03	88.45 ± 0.02
DuA (Pan Y. et al., 2024)	95.53 ± 0.02	90.87 ± 0.03	91.23 ± 0.02	85.25 ± 0.01	91.63 ± 0.03	87.13 ± 0.02	90.80 ± 0.01	87.29 ± 0.03
Ours	97.23 ± 0.01	94.68 ± 0.03	93.89 ± 0.01	95.74 ± 0.03	98.43 ± 0.02	93.90 ± 0.02	92.34 ± 0.03	96.24 ± 0.01

## 5 Conclusion and discussion

The primary goal of this study is to address the complex challenges in sports performance analysis, particularly in real-time monitoring and recognition of movements by integrating electroencephalogram (EEG) signals and video data. Traditional methods often fall short when handling multimodal data, especially in capturing cross-modal dependencies and ensuring real-time processing. To this end, we propose a novel EEG-driven model called the “Cerebral Transformer.” This model effectively integrates EEG signals and video data through adaptive attention mechanisms and cross-modal fusion for precise analysis of sports performance. In our experiments, we validated the model using the SEED, DEAP, eSports Sensors, and MODA datasets. The results showed that our model outperformed six state-of-the-art (SOTA) models in terms of accuracy, recall, and F1 score. Additionally, ablation studies revealed that the cross-modal attention mechanism and adaptive attention mechanism significantly impact the model's performance, especially in efficiently processing the fusion of EEG signals and video data. Our approach achieved faster inference and training times, maintaining low parameter count and minimal floating-point operations, making it suitable for real-time sports monitoring scenarios.

Despite the significant experimental results, the model still has some limitations. We acknowledge several limitations in our study that warrant further discussion. First, the datasets used in our experiments, such as SEED, DEAP, eSports Sensors, and MODA, may introduce inherent biases due to their specific experimental setups and participant demographics. These biases could limit the generalizability of our model to broader populations or diverse real-world scenarios. For instance, the SEED and DEAP datasets primarily include controlled laboratory settings, which may not fully capture the variability of real-world conditions. Second, while our model is designed to process multimodal data effectively, real-time application poses significant challenges. These include the need for low-latency data processing, robust handling of noisy or incomplete signals, and ensuring computational efficiency on resource-constrained devices. Although we evaluated computational metrics such as inference time and FLOPs, further work is needed to optimize the model for real-time deployment without compromising accuracy. Lastly, while we demonstrated the efficacy of our model on a range of tasks, additional evaluation on larger and more diverse datasets, as well as under real-world conditions, is necessary to confirm its robustness and reliability. Future work could address these limitations by incorporating more diverse datasets, exploring domain adaptation techniques, and optimizing the model for deployment on edge devices.

## Data Availability

The original contributions presented in the study are included in the article/supplementary material, further inquiries can be directed to the corresponding author.
